# *Rice stripe virus* NS3 protein regulates primary miRNA processing through association with the miRNA biogenesis factor OsDRB1 and facilitates virus infection in rice

**DOI:** 10.1371/journal.ppat.1006662

**Published:** 2017-10-04

**Authors:** Lijia Zheng, Chao Zhang, Chaonan Shi, Zhirui Yang, Yu Wang, Tong Zhou, Feng Sun, Hong Wang, Shanshan Zhao, Qingqing Qin, Rui Qiao, Zuomei Ding, Chunhong Wei, Lianhui Xie, Jianguo Wu, Yi Li

**Affiliations:** 1 State Key Laboratory of Ecological Pest Control for Fujian and Taiwan Crops, Fujian Province Key Laboratory of Plant Virology, Institute of Plant Virology, Fujian Agriculture and Forestry University, Fuzhou, China; 2 The State Key Laboratory of Protein and Plant Gene Research, College of Life Sciences, Peking University, Beijing, China; 3 Institute of Plant Protection, Jiangsu Academy of Agricultural Sciences, Nanjing, China; Agriculture and Agri-Food Canada, CANADA

## Abstract

MicroRNAs (miRNAs) are small regulatory RNAs processed from primary miRNA transcripts, and plant miRNAs play important roles in plant growth, development, and response to infection by microbes. Microbial infections broadly alter miRNA biogenesis, but the underlying mechanisms remain poorly understood. In this study, we report that the *Rice stripe virus* (RSV)-encoded nonstructural protein 3 (NS3) interacts with OsDRB1, an indispensable component of the rice (*Oryza sativa*) miRNA-processing complex. Moreover, the NS3-OsDRB1 interaction occurs at the sites required for OsDRB1 self-interaction, which is essential for miRNA biogenesis. Further analysis revealed that NS3 acts as a scaffold between OsDRB1 and pri-miRNAs to regulate their association and aids *in vivo* processing of pri-miRNAs. Genetic evidence in *Arabidopsis* showed that NS3 can partially substitute for the function of double-stranded RNA binding domain (dsRBD) of AtDRB1/AtHYL1 during miRNA biogenesis. As a result, NS3 induces the accumulation of several miRNAs, most of which target pivotal genes associated with development or pathogen resistance. In contrast, a mutant version of NS3 (mNS3), which still associated with OsDRB1 but has defects in pri-miRNA binding, reduces accumulation of these miRNAs. Transgenic rice lines expressing *NS3* exhibited significantly higher susceptibility to RSV infection compared with non-transgenic wild-type plants, whereas the transgenic lines expressing *mNS3* showed a less-sensitive response. Our findings revealed a previously unknown mechanism in which a viral protein hijacks OsDRB1, a key component of the processing complex, for miRNA biogenesis and enhances viral infection and pathogenesis in rice.

## Author summary

MicroRNAs (miRNAs) regulate gene expression at the transcriptional or post-transcriptional level and have emerged as key players in regulating plant growth, development and response to biotic and abiotic stresses. Accumulating evidences suggest that miRNAs are pivotal modulators of host–virus interactions, but how virus regulates miRNA accumulation remains poorly understood. Here, we report that NS3 protein encoded by *Rice stripe virus* (RSV) regulates the processing of several primary miRNA transcripts (pri-miRNAs) by acting as an intermediary to modulate the association of pri-miRNAs and OsDRB1, a key factor of the pri-miRNA processing complex. NS3 increases recruitment of pri-miRNA to the processing complex by its association with OsDRB1 at the sites required for OsDRB1 dimer formation and induces several miRNAs accumulations as well as target genes repression, promoting the sensitivity of rice to RSV infection. Together these findings reveal a novel mechanism by which RSV regulates pri-miRNA processing, leading to enhanced viral infection.

## Introduction

MicroRNAs (miRNAs), a class of endogenous small RNAs processed from their primary transcripts (pri-miRNAs), are crucial for plant development and responses to abiotic and biotic stresses [1–3]. Invading pathogens can manipulate the biogenesis and stability of many miRNAs to promote infection, or affect plant defense. For example, the accumulation of miR168 is elevated by infections with *Cymbidum ringspot virus* (CymRSV), *crucifer-infecting Tobacco mosaic virus* (crTMV), *Potato virus X* (PVX), and *Tobacco etch virus* (TEV) in *Nicotiana benthamiana* and by *Rice stripe virus* (RSV) and *Rice dwarf virus* (RDV) in rice (*Oryza sativa*). Indeed, this induced accumulation was found to promote the infection process of these viruses [4–9]. *Rice ragged stunt virus* (RRSV) and *Rice black streaked dwarf virus* (RBSDV) infections in rice increase the level of miR319, which suppresses jasmonic acid mediated antiviral defense in rice [10]. Overexpression of miR528 in transgenic rice plants reduces the accumulation level of reactive oxygen species (ROS) compared with that of wild type (WT), and these plants are more sensitive to RSV infection [11]. *Arabidopsis* miR393 suppresses auxin signaling in response to bacterial infection [12], while miR398b is involved in defense against fungal pathogens [13]. In addition, some miRNAs that function in basal metabolism are regulated by pathogen invasion. For instance, miR395 and miR399, which are involved in the regulation of sulfur assimilation [14,15] and response to phosphorus starvation [16–18], respectively, are up-regulated by RSV infection in rice [19].

In plants, the miRNA biogenesis pathway is regulated by the cooperation of several host factors [20]. Work in *Arabidopsis* showed that miRNAs are processed from primary transcripts that contain partially complementary fold-back regions of variable lengths (pri-miRNAs) by a processing complex consisting of the RNAse III enzyme DICER-LIKE 1 (AtDCL), the double-stranded RNA (dsRNA) binding protein HYPONASTIC LEAVES1 (AtDRB1/AtHYL1), and the zinc finger protein SERRATE (AtSE) [21–23]. AtHYL1 and AtSE are essential for the accurate and efficient cleavage of pri-miRNAs by AtDCL1 [21]. Homodimerization/self-interaction of AtHYL1 (orthologous to the OsDRB1) ensures the correct selection of cleavage sites on pri-miRNAs [24]. After processing, the miRNA/miRNA* duplex is methylated by HUA ENHANCER 1 (AtHEN1) at the 3’-terminus to ensure the stability of mature miRNA [25,26]. The miRNA strand is recruited by diverse ARGONAUTE (AtAGO) proteins to form miRNA-induced silencing complexes to mediate post-transcription gene silencing or translation repression [27–29]. Although the miRNA biogenesis pathway in plants has been well documented and many studies have indicated that miRNAs are involved in host–virus interactions, little is known about how pathogens regulate miRNA processing and accumulation.

*Rice stripe virus* (RSV), the type member of the genus *Tenuivirus*, causes severe disease and yield losses in many Asian rice cultivars. The RSV genome comprises four negative-sense, single-stranded RNA segments, RNA1, 2, 3, and 4. RNA1 uses a negative sense coding strategy while RNA2, 3, and 4 use ambisense coding strategy [30–33]. RNA1 encodes RNA-dependent RNA polymerase (RdRp, 337 kDa) [34]. RNA2 encodes NS2 (22.8 kDa), a weak suppressor of RNA silencing [35] and NSvc2 (94.2 kDa), a glycoprotein that targets the Golgi body and the endoplasmic reticulum (ER) [36]. RNA3 encodes nonstructural protein 3 (NS3, 23.9 kDa) and the coat protein (CP, 35.1 kDa) [37,38]. NS3 was identified as a viral-encoded RNA silencing suppressor (VSR) which suppresses post-transcriptional gene silencing (PTGS) in *N*. *benthamiana* and binds single- or double-stranded RNA without sequence preference [37,38]. RNA4 encodes disease specific protein (SP, 20.5 kDa) which interferes with photosynthesis by interaction with an oxygen-evolving complex protein [30] and NSvc4 (32.4 kDa), a cell-to-cell movement protein [39]. Several studies have demonstrated that RSV infection perturbs miRNA accumulation, for example, 38 miRNAs including miR167, miR168, miR395, miR399 etc., were induced upon RSV infection between 7 to 15 dpi [9,19], but the underlying mechanism for this is unclear. In this study, we found that RSV-encoded NS3 is responsible for the over-accumulation of several miRNAs, many of them known to regulate biotic or abiotic stress response genes, in a dsRNA-binding activity-dependent manner both in rice and *Arbidopsis*. *In vivo* experiments demonstrated that NS3 enhanced pri-miRNA processing through its dsRNA binding domain. Additionally, NS3 interacts specifically with the second dsRNA-binding domain (dsRDB2) of OsDRB1. Importantly, the NS3-interacting sites in OsDRB1 are also required for the homodimerization of the OsDRB1 and NS3 acts as a scaffold to regulate the association of OsDRB1 and pri-miRNA. Genetic analysis showed that NS3-ΔdsRBD-AtHYL1 fusion protein could partially rescue the phenotype of *hyl1-2*, but NS3 or ΔD1-AtHYL1 could not do so. *NS3*-overexpressing transgenic rice lines enhanced RSV pathogenicity compared with the control transgenic lines expressing a mutant form of *NS3* (mNS3) and the WT plants. Our data revealed that the RSV NS3 protein regulates the association between OsDRB1 and pri-miRNAs, induces accumulation of a number of miRNAs, and enhances viral pathogenicity in rice.

## Results

### NS3 is responsible for the RSV-induced over-accumulation of a set of miRNAs

**Fig 1:**
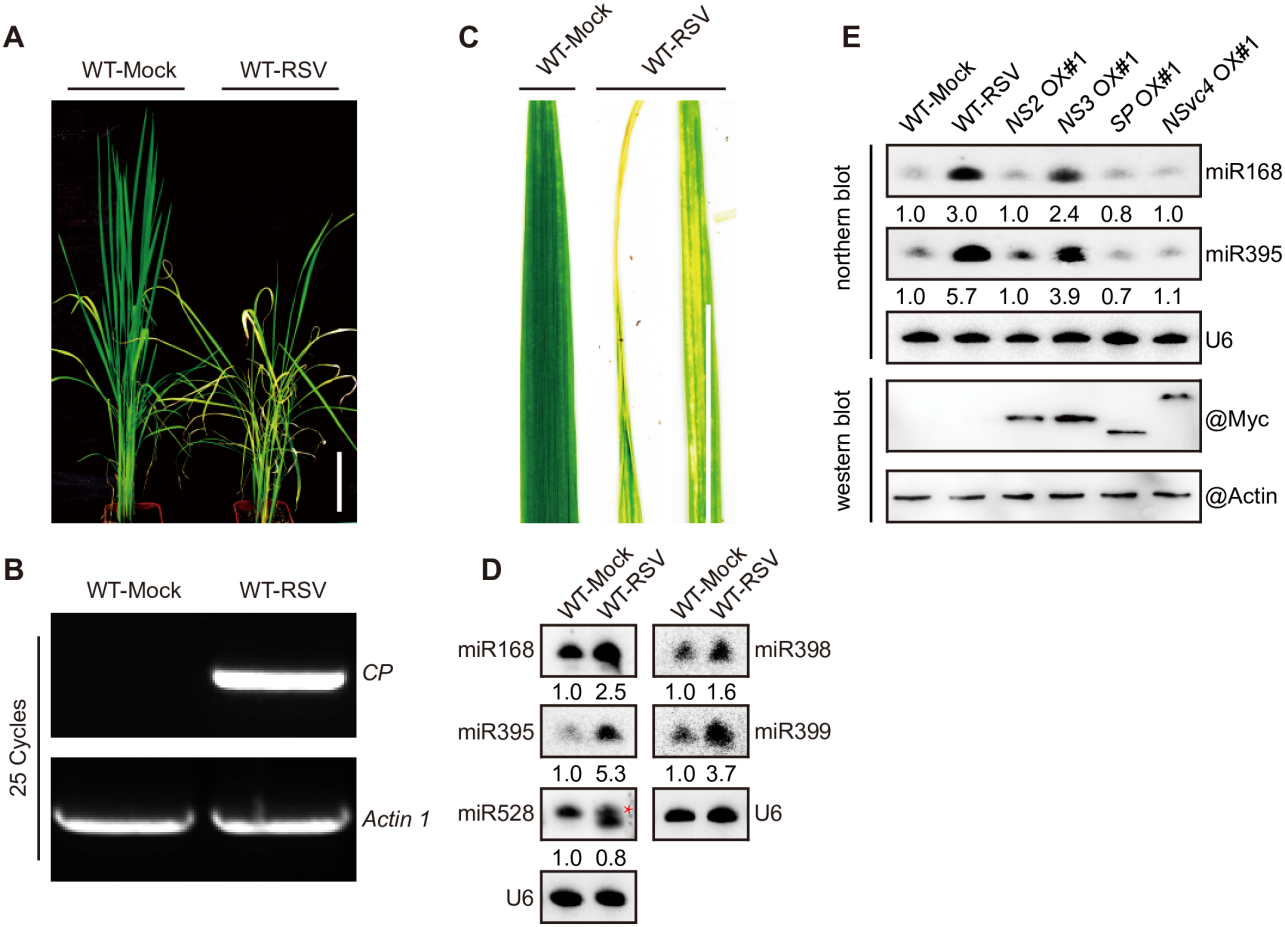
RSV symptoms and miRNAs induced by NS3 in rice. (A) Images of whole plants exhibiting stunted phenotypes. Scale bar = 15 cm. (B) Detection of the RSV *CP* gene by RT-PCR. (C) Images of RSV-infected leaves exhibiting chlorotic mottling and rolled-leaf phenotypes. Scale bar = 5 cm. (D) Detection of miRNA (miR168, 395, 398, 399, and 528) accumulations in healthy (mock-infected) and RSV-infected rice plants by northern blotting. Red star, 21-nt miR528. (E) Northern and western blot assay detection of miRNA (miR168 and miR395) accumulations and protein expression in mock-infected, RSV-infected (RSV), *NS2* OX#1, *NS3* OX#1, *SP* OX#1, and *NSvc4* OX#1 rice plants. In (D and E), U6 served as a loading control, the expression levels in the WT-Mock plants are set to a value of 1.0 and the expression levels in the other plants are relative to this reference value.

RSV-infected rice plants show stunting, rolled-leaf and chlorotic mottling in leaves symptom compared to mock-infected rice plants at early stage (Fig 1A to 1C). In a previous study, we found that RSV infection resulted in an increased accumulation of several rice miRNAs, including miR168, miR395, miR398, miR399 and miR528 (20-nt and 21-nt forms) [5,9,11,19]. To confirm the RSV-induced increase in the accumulation of these miRNAs in a different set of plants, we analyzed the differential expression of these rice miRNAs in mock-inoculated and RSV-infected rice plants by northern blotting. All tested miRNAs, with the exception of miR528 (21-nt), were up-regulated by RSV infection (Fig 1D), which was consistent with previous reports [5,11,40,41].

To determine which RSV-encoded protein triggers the up-regulation of miRNAs in the RSV-infected rice plants, we measured the expression of these miRNAs in rice plants overexpressing various RSV-encoded proteins, driven by the *ACTIN 1* promoter, with a 4×Myc epitope tag at the N-terminus. Western blot assays confirmed the expression of myc-NS2, myc-NS3, myc-SP, and myc-NSvc4 in the corresponding transgenic rice lines (Fig 1E) (bottom two panels). As shown in Fig 1E, (top three panels), the levels of miR168 and miR395, measured by northern blot hybridizations, were strongly elevated by RSV infection and transgenic expression of NS3 (Fig 1E, lane 2 and 4), but not other RSV proteins. Also, RSV infection and NS3 transgene expression is produced similar elevations of miRNA levels (Fig 1E, lane 2 and 4). Therefore, expression of NS3 alone fully recapitulates the perturbation of miRNA levels caused by RSV infections.

### NS3 triggers the accumulation of several miRNAs through its dsRNA binding domain

**Fig 2:**
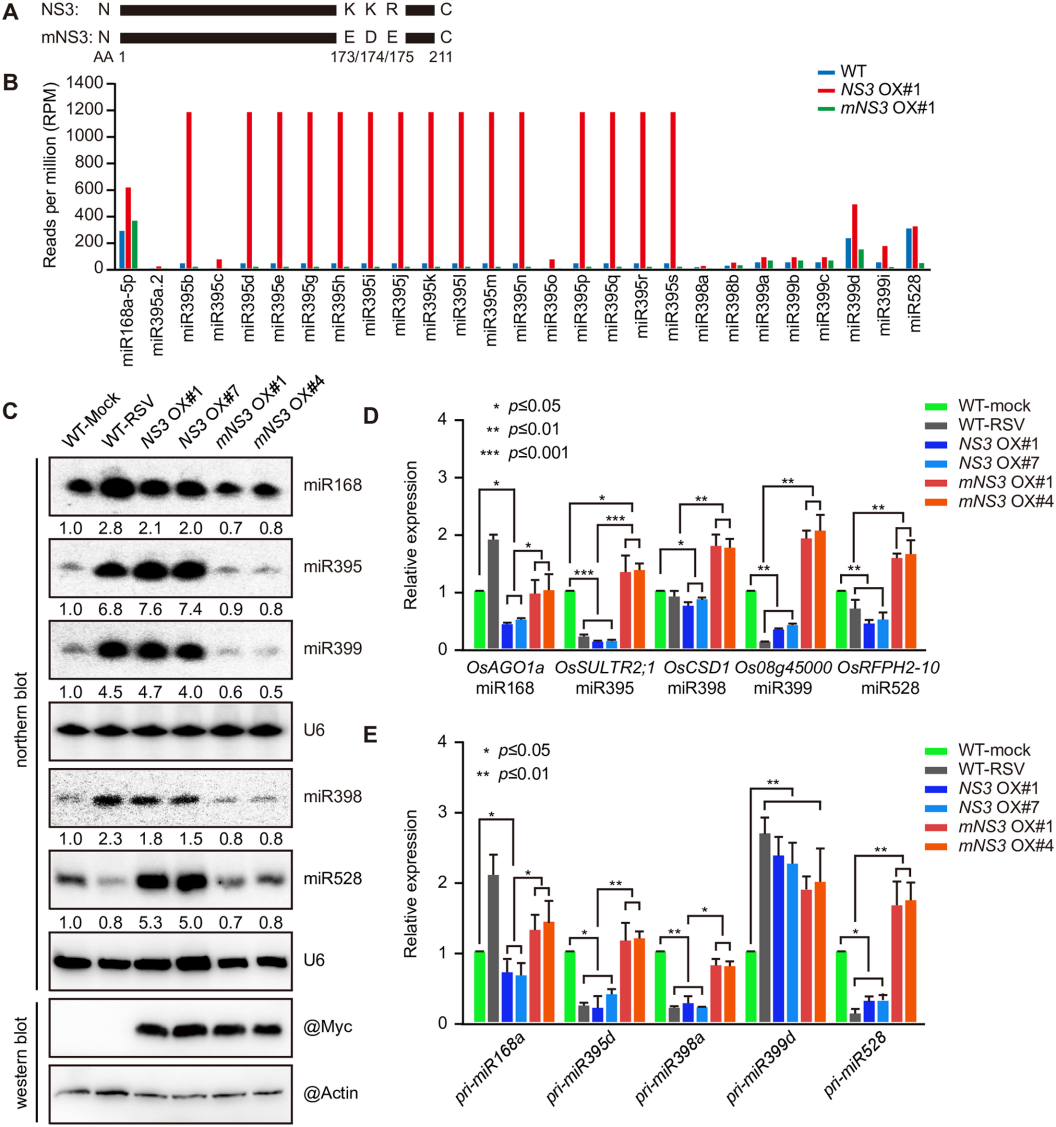
Expression of NS3 promotes the accumulation of several miRNAs and reduces the expression of their targets. (A) Mutation site of the mutant NS3 (mNS3). (B) Measurement of miRNA (miR168, 395, 398, 399, and 528) accumulation in the WT, *NS3* OX#1, and *mNS3* OX#1 rice lines by small RNA sequencing. (C) Detection of miRNA (miR168, 395, 398, 399, and 528) accumulations in mock-infected, RSV-infected (RSV), *NS3* OX#1, *NS3* OX#7, *mNS3* OX#1, and *mNS3* OX#4 rice plants by northern blotting. U6 served as a loading control, the expression levels in the WT-Mock plants are set to a value of 1.0 and the expression levels of in the other plants are relative to this reference value. (D) Relative expression levels of the target genes of the miRNAs (miR168, 395, 398, 399, and 528), including *OsAGO1a*, *OsSULTR2;1*, *OsCSD1*, *Os08g45000*, and *OsRFPH2-10* in the mock-infected, RSV-infected (RSV), *NS3* OX#1, *NS3* OX#7, *mNS3* OX#1, and *mNS3* OX#4 rice plants. (E) Relative expression levels of the miRNA (miR168, 395, 398, 399, and 528) precursors, including *pri-miR168a*, *pri-miR395d*, *pri-miR398a*, *pri-miR399b*, and *pri-miR528* in the mock-infected, RSV-infected (RSV), *NS3* OX#1, *NS3* OX#7, *mNS3*OX#1, and *mNS3* OX#4 rice plants. Average (± SD) values based on RT-qPCR analysis of three biological replicates are shown. ***, *P* ≤ 0.001; **, *P* ≤ 0.01; *, *P* ≤ 0.05.

The dsRNA binding domain of NS3 is important for its activity [37]. To determine whether NS3-promoted miRNA accumulation depends on its dsRNA binding activity, we constructed a transgenic rice line overexpressing Myc-tagged *mNS3*, in which the dsRNA binding domain was disrupted by the replacement of a 173K174K175R motif with173E174D175E (Fig 2A). We then conducted *de novo* sequencing of small RNAs in the WT, and the *NS3* overexpression (OX) #1 and *mNS3* OX#1 rice lines. The results showed that miR168, miR395, miR398, miR399, and miR528 were all up-regulated in the *NS3*-overexpressing rice line, but down-regulated or unchanged in the *mNS3*-overexpressing rice line compared with the WT (See S1 Table for more details) (Fig 2B). To verify the *de novo* sequencing results, we carried out northern blot and western blot assays in two independent overexpression lines each for *NS3* and *mNS3* (*NS3* OX#1 and #7 and *mNS3* OX#1 and #4). As shown in Fig 2C, most miRNAs accumulated at higher levels in the *NS3* overexpression lines compared with the mock-inoculated WT, whereas lower levels were detected in the *mNS3* overexpression lines. These results indicated that NS3 plays a critical role in the positive regulation of miRNA accumulations in rice and this regulation is dependent on its dsRNA binding activity.

To verify that the increased accumulation of these miRNAs further reduced the expression levels of their target genes, we examined the expression levels of their target mRNAs by quantitative RT-PCR (RT-qPCR). As shown in Fig 2D, mRNA levels of *OsAGO1a* (miR168), *OsSULTR2;1* (miR395), *OsCDS1* (miR398), *Os08g45000* (miR399) and *OsRFPH2-10* (miR528) were all reduced in the *NS3* overexpression lines compared with the mock-inoculated WT plants, whereas expression levels in the *mNS3* overexpression lines were relatively unchanged compared to the WT.

To further test whether NS3 triggers accumulation of miRNAs through its dsRNA binding domain in dicots, we constructed *NS3* and *mNS3* overexpression transgenic *Arabidopsis* plants. Northern blot showed that the levels of miR168 and miR395 were up-regulated by RSV infection and *NS3* overexpression in these *Arabidopsis* plants (S1A and S1B Fig). Although overexpression of *NS3* in rice did not result in disease-like symptoms, *NS3*-overexpressing *Arabidopsis* plants exhibited a severely stunted phenotype that phenocopied the disease symptoms of the RSV infected *Arabidopsis* (S1C and S1D Fig). In contrast, overexpression of *mNS3* had no influence on the growth or the levels of miR168 and miR395 in the *Arabidopsis* plants (S1B and S1D Fig). These results indicate that induction of miRNA accumulations by *NS3* overexpression is conserved in dicots and monocots.

To determine whether RSV infection and *NS3* overexpression increases the accumulation of these miRNAs through promoting primary miRNA (pri-miRNA) processing, we measured the primary transcript levels of miR168, miR395, miR398, miR399, and miR528 by RT-qPCR. The results demonstrated that NS3, but not mNS3, down-regulated all of the pri-miRNAs, except for pri-miR399d, which suggests that NS3 is involved in pri-miRNA processing in a dsRNA-binding activity-dependent manner (Fig 2E).

### NS3 aids *in vivo* processing of pri-miRNA

**Fig 3:**
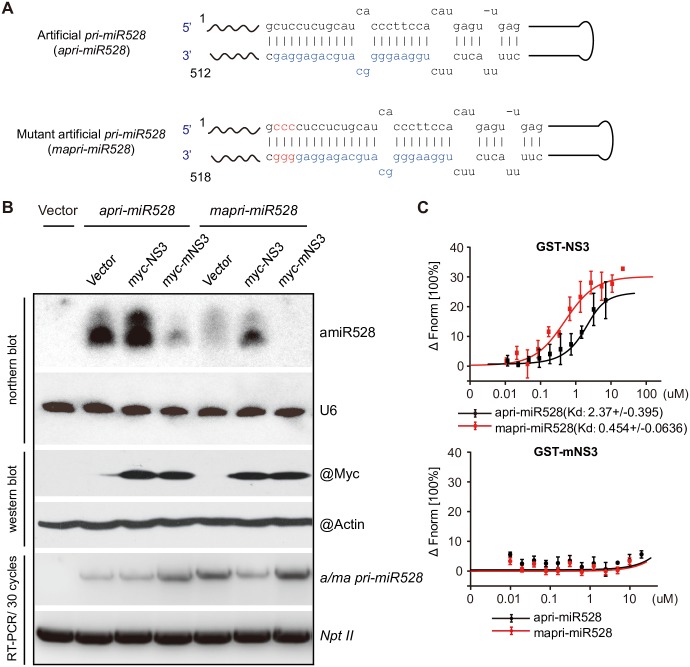
NS3 promotes pri-miRNA processing. (A) Structures of artificial *pri-miR528* (*apri-miR528*) and mutated artificial *pri-miR528* (*mapri-miR528*). (B) Northern blot, western blot, and RT-PCR detection of the products of transiently co-expressed pri-miRNAs (apri-miR528 or mapri-miR528) and proteins (empty vector, NS3, or mNS3) in *N*. *benthamiana*. (C) Results of a microscale thermophoresis assay shows the interactions between pri-miRNA (apri-miR528 or mapri-miR528) and protein (GST-NS3 or GST-mNS3).

Given that NS3 reduces the accumulation of a set of pri-miRNAs but induces the accumulation of corresponding mature miRNAs, we suggested that NS3 may promote the recruitment of pri-miRNAs by affecting the miRNA-processing complex. Since the thermo stability of the end of the miRNA/miRNA* duplex is important for mature miRNA accumulation [42], we designed an experiment in which a pri-miRNA can be recognized by NS3, but not by the processing complex, which would indicate that NS3 directly assists in the association of the processing complex with pri-miRNAs when *NS3* is co-expressed with the pri-miRNA. We constructed a 35S promoter-driven artificial *primary miR528* (*apri-miR528*) and mutant artificial *primary miR528* (*mapri-miR528*) with three additional C/G pairs at the end of the miRNA/miRNA* duplex using an *Arabidopsis primary miR159a* backbone (Fig 3A). miR528 is only expressed in monocot plants; therefore, we carried out an *in vivo* transient expression assay to co-express *apri-miR528* and *vector/NS3/mNS3* and also *mapri-miR528* and *vector/NS3/mNS3* in the leaves of the dicots *N*. *benthamiana* and measured mature artificial miR528 (amiR528) levels in each group by northern blotting at 3 days post-infiltration (dpi). We found that expression of *NS3* had little influence on the accumulation of amiR528 in the *apri-miR528* group, but expression of*mNS3* reduced the levels of miR528 relative to the vector control. As expected, no miR528 accumulation was observed in the negative control. Additionally, no mature miR528 accumulation was detected in the *mapri-miR528* group in the absence of *NS3* expression. The accumulation of mature miRNA was only detected in the leaves co-expressing *mapri-miR528* with *NS3* (Fig 3B). To test if NS3 interacts with apri-miR528 and mapri-miR528, an *in vitro* microscale thermophoresis assay with GST-mNS3 serving as the negative control was used to reveal that both apri-miR528 and mapri-miR528 were recognized by GST-NS3 (Fig 3C). These results provide further evidence for the role of NS3 in mature miRNA biogenesis.

### Association of NS3 with the dsRBD2 domain of OsDRB1

**Fig 4:**
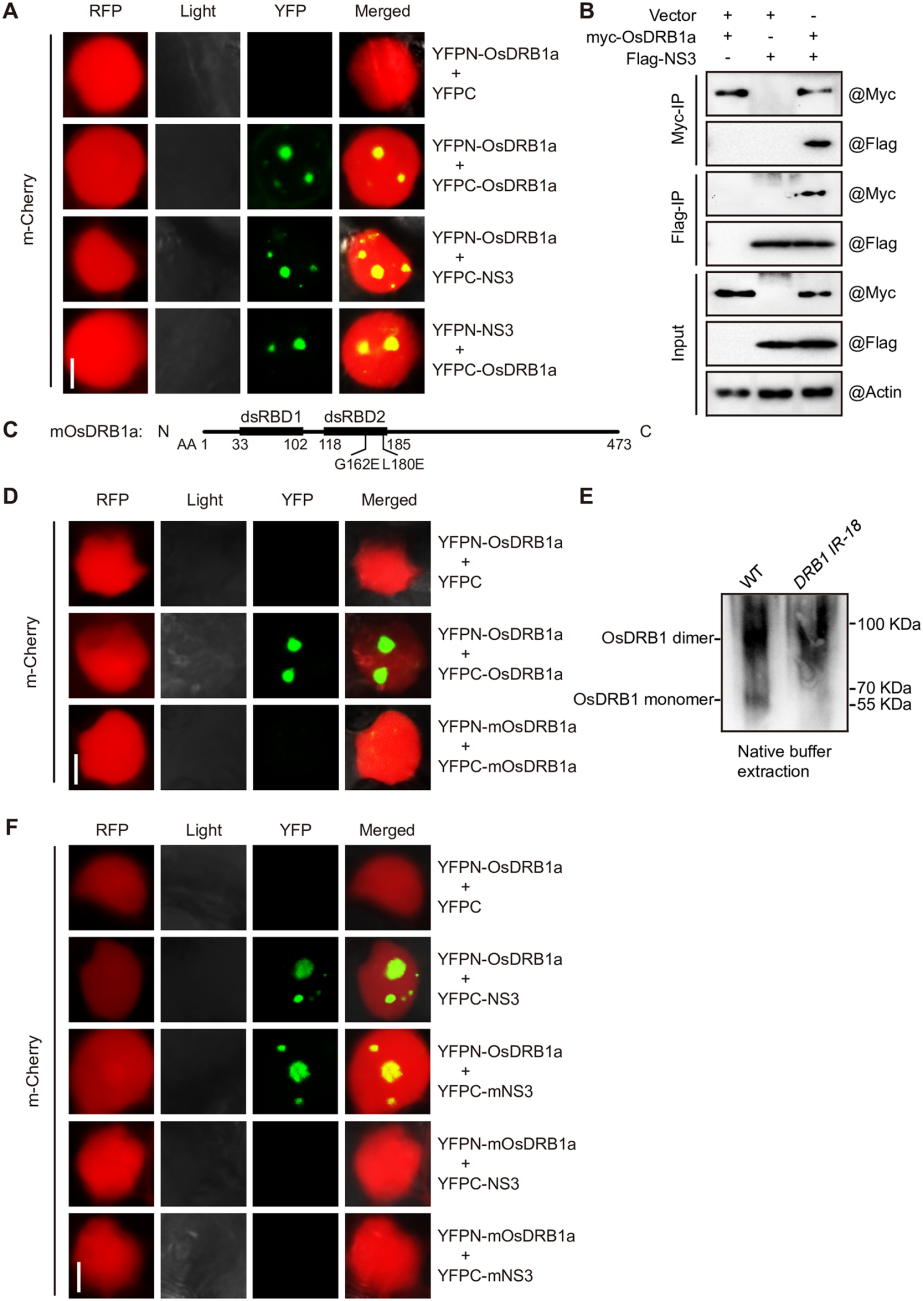
NS3 interacts with OsDRB1, a pri-miRNA processing factor. The BiFC assay was conducted in *N*. *benthamiana* epidermal cells, mCherry is a nuclear localization marker fused with red florescent protein (RFP). (A) Results of a BiFC assay showing the interaction between NS3 and OsDRB1a. Scale bar = 0.1 μm. (B) Results of a co-immunoprecipitation analysis showing the interaction between NS3 and OsDRB1a. (C) The accuracy of mutation sites of mOsDRB1a. (D) Results of a BiFC assay showing the accuracy of interaction sites between OsDRB1a. Scale bar = 0.1 μm (E) Formation of OsDRB1a dimers *in vivo*. Total rice protein extracts from the WT and *OsDRB1*-knockdown lines were treated with ‘‘native” buffer and detected using anti-HYL1antibodies. (F) Results of a BiFC assay showing the accuracy of interaction sites between NS3 and DRB1a. Scale bar = 0.1 μm.

NS3 does not contain an RNase III domain; therefore, it cannot promote miRNA accumulation by itself. We hypothesized that NS3 may function in miRNA processing through its association with components of the Dicing body (D-body) to promote the recruitment of the pri-miRNA by the processing complex. To test this hypothesis, we performed bimolecular fluorescence complementation (BiFC) assays to co-express *Arabidopsis DCL1* (*AtDCL1*), *SE* (*AtSE*), *HYL1* (*AtHYL1*), or *CBP20* (*AtCBP20*) with *NS3* in *N*. *benthamiana* leaves. NS3 associated with AtHYL1 and AtSE but not with AtDCL1 or AtCPB20. In addition, we found that AtHYL1, but not AtSE, specifically interacted with NS3 in the D-body, and the interaction between NS3 and AtHYL1 may involve in miRNA maturation (S2A Fig). Using the basic local alignment search tool (BLAST) and the UniProt protein database (uniprot.org), we found six AtHYL1 homologs in rice, OsDRB1a, OsDRB1b, OsDRB1c, OsDRB2, OsDRB3 and OsDRB4 (S2B Fig).To confirm that OsDRB1 has the same function as AtHYL1, we measured the levels of miR164, miR166, and miR168 by small-RNA RT-qPCR in an *OsDRB1*-knockdown rice line and found that these miRNAs were down-regulated (S2C Fig). We also analyzed OsDRB1 protein levels, and found that the level of OsDRB1 was reduced in the *OsDRB1*-knockdown line (S2D Fig). OsDRB1a contains all the domains of the other two OsDRB1s, as well as a unique C-terminus, so we chose OsDRB1a to test the interaction between NS3 and OsDRB1, BiFC and co-immunoprecipitation (CoIP) assays demonstrated that OsDRB1a does interact with NS3 (Fig 4A and 4B). We also tested interactions between NS3 and the other OsDRBs by BiFC assay, and found that only OsDRB2 has a weak interaction with NS3 in cytoplasm (S2E Fig). Previous studies have shown that homodimerization of AtHYL1 is required for AtDCLs to locate the correct cleavage sites in pri-miRNAs, while the G147 and L165 residues of AtHYL1 are critical for homodimer formation [24]. The G162 and L180 residues of OsDRB1a correspond to the G147 and L165 residues of AtHYL1 and may be essential for homodimer formation according to amino acid alignment (S2F Fig). To test our hypothesis, we constructed an *OsDRB1a* mutant (*mOsDRB1a*) by replacing the G162 and L180 residues with E162 and E180, respectively (Fig 4C). In a subsequent BiFC assay, we found that mOsDRB1a could not interact with itself (Fig 4D), as expected. We further confirmed that wild-type OsDRB1a, like AtHYL1, formed a homodimer (Fig 4E) [24,43]. Also, mOsDRB1a could not interact with NS3 or mNS3 (Fig 4F), but NS3 interacts with itself, mNS3 has weak interaction with NS3, and OsDRB1a has weak interaction with mOsDRB1a too (S2G Fig). We summarize the interaction of each pair between NS3, mNS3, OsDRB1a and mOsDRB1a in Table 1. These results demonstrated that the association between NS3 and OsDRB1a depends on the dsRBD2 domain of OsDRB1a and is essential for miRNA processing.

**Table 1:** Interactions of each two proteins as shown below.

	NS3	mNS3	OsDRB1a	mOsDRB1a
NS3	√√	√	√√	×
mNS3	√	×	√√	×
OsDRB1a	√√	√√	√√	√
mOsDRB1a	×	×	√	×

^√√:^ strong interaction;

^√:^ weak interaction;

^×:^ no interaction.

### NS3 mimics the dsRBD domain of DRB1 in miRNA processing

**Fig 5:**
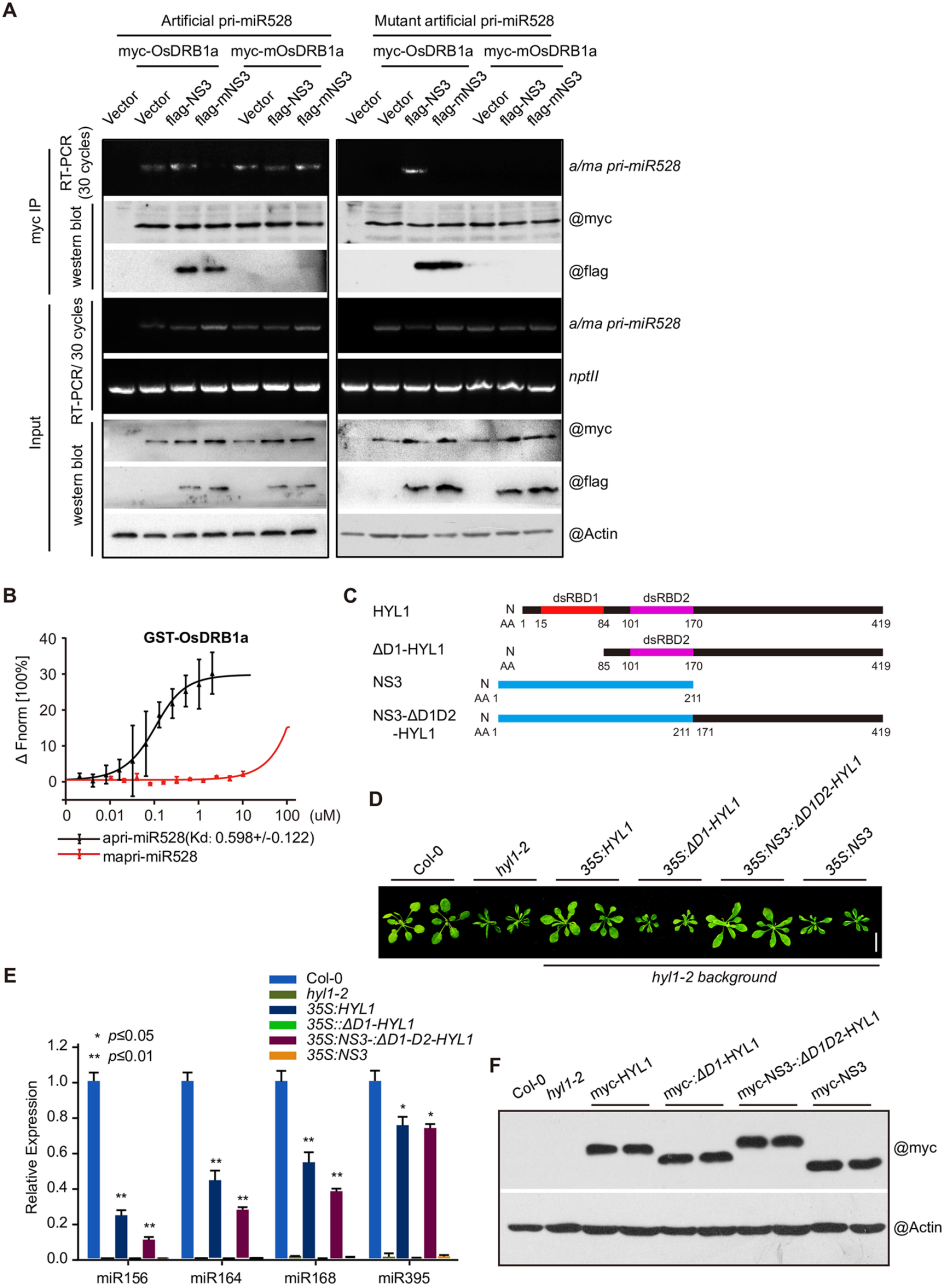
NS3 acts as a scaffold between DRB1 and pri-miRNA. (A) RT-PCR and western blot detection of co-immunoprecipitated and input products of transiently co-expressed protein (DRB1a or mDRB1a), pri-miRNA (apri-miR528 or mapri-miR528), and protein (empty vector, NS3, or mNS3) in *N*. *benthamiana*. (B) Results of a microscale thermophoresis assay shows the interactions between pri-miRNA (apri-miR528 or mapri-miR528) and GST-OsDRB1a. (C) Gene structure of *HYL1*, Δ*D1-HYL1*, *NS3* and *NS3*-Δ*D1D2-HYL1*. (D) Phenotype of Col-0, *hyl1-2* and transgenic *Arabidopsis* plants overexpressed *AtHYL1*, Δ*D1-HYL1*, *NS3*-Δ*D1D2-HYL1* and *NS3* with the 35S promoter in the *hyl1-2* mutant background. (E) miRNA (miR156, miR164, miR168 and miR395) levels in Col-0, *hyl1-2* and transgenic *Arabidopsis* plants overexpressing *AtHYL1*, Δ*D1-HYL1*, *NS3*-Δ*D1D2-HYL1* and *NS3* with the 35S promoter in the *hyl1-2* mutant background. (F) Western blot of AtHYL1, ΔD1-HYL1, NS3-ΔD1D2-HYL1 and NS3 transgenic *Arabidopsis* plants. Fnorm, normalized fluorescence.

Given that the dsRNA-binding activity of NS3 is important for the processing of pri-miRNAs and the accumulation of miRNA, and NS3 interacts with DRB1, we deduced that NS3, rather than DRB1, recognizes pri-miRNAs during NS3–DRB1 interactions. To test this hypothesis, we transiently co-expressed *OsDRB1a/mOsDRB1a*, *apri-miR528/mapri-miR528*, or *empty vector/NS3/mNS3* in *N*. *benthamiana* and detected the CoIP products of *Os*DRB1a/m*Os*DRB1a by RT-PCR. We found that both OsDRB1a and mOsDRB1a associated with apri-miR528, but neither of them recognized mapri-miR528, and NS3 and mNS3 associated with OsDRB1a instead of mOsDRB1a. With the expression of *NS3*, both OsDRB1a and mOsDRB1a associated with apri-miR528. However, with the expression of *mNS3*, only mOsDRB1a associated with apri-miR528. Additionally, only co-expression with *NS3* resulted in an OsDRB1a interaction with mapri-miR528 (Fig 5A), see Table 2 for more details. Using a microscale thermophoresis assay, we also found that OsDRB1a associated with apri-miR528 but not mapri-miR528 (Fig 5B). We also found that NS3, but not mNS3, could bind with the endogenous miRNA precursors (pre-miR168a, pre-miR395d, pre-miR398a, pre-miR399d, and pre-miR528) in an electrophoretic mobility shift assay (EMSA) (S3A Fig). These results indicated that by interacting with DRB1, NS3 replaced the dsRNA-binding activity of DRB1. To test this hypothesis, we overexpressed *AtHYL1*, Δ*D1-HYL1*, a double-stranded RNA binding domain 1 deletion form of *AtHYL*, *NS3*-Δ*D1D2-HYL1*, a fusion protein of NS3 and ΔD1D2-HYL1 and *NS3* (Fig 5C) in *Arabidopsis hyl1-2* mutant background, the transgenes were all driven by a 35S promoter with the proteins tagged with a myc-epitope tag at the N-terminus, we found that the fusion protein NS3-ΔD1D2-AtHYL1 could ameliorated the phenotype (Fig 5D) and miRNA (miR156, miR164, miR168 and miR395) levels (Fig 5E) of *hyl1-2* mutant as HYL1 did but NS3 or ΔD1-HYL1 could not (Fig 5D and 5E), we test these transgenic *Arabidopsis* plants by western blotting (Fig 5F), and this results indicated that NS3 could substitute for the dsRBD domain of AtHYL1 in miRNA processing.

**Table 2:** Interaction of apri-miR528 or mapri-miR528 with single protein or protein complex as shown below.

	NS3	mNS3	OsDRB1a	OsDRB1a+NS3	OsDRB1a+NS3	mOsDRB1a	mOsDRB1a+NS3	mOsDRB1a+NS3
apri-miR528	√	×	√	√	×	√	√	√
mapri-miR528	√	×	×	×	×	×	√	×

^√:^ Yes;

^×:^ No.

### NS3 enhances virus pathogenicity in rice

**Fig 6:**
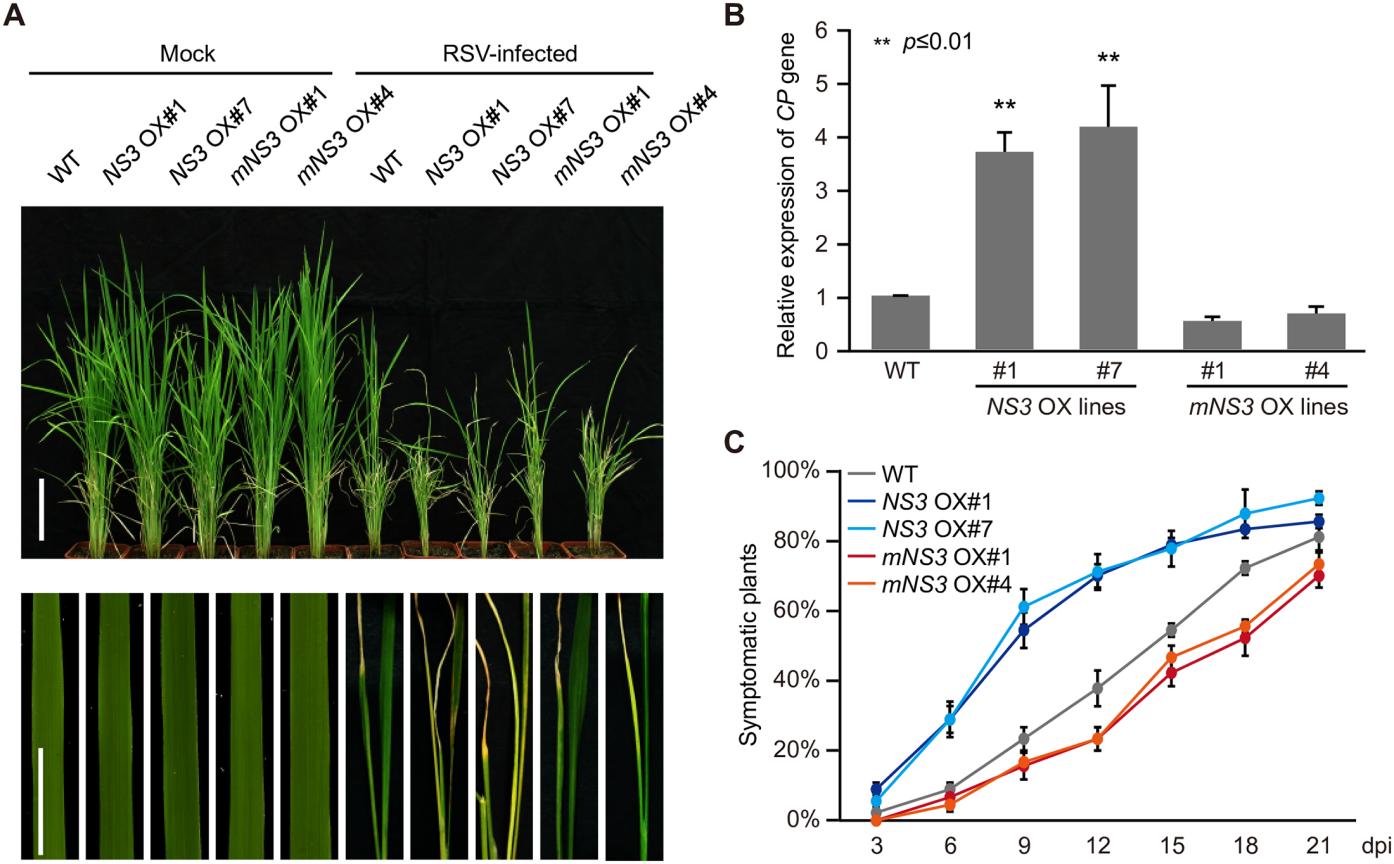
Disease symptoms induced by RSV infection in WT, *NS3* OX, and *mNS3* OX rice lines. (A) Images of whole plants and details showing stunted or folded-leaf phenotypes of the wild-type, *NS3* OX#1, *NS3* OX#7, *mNS3* OX#1 and *mNS3* OX#4 rice plants. Scale bars = 15 cm (upper panel) and 5 cm (lower panel). (B) Detection of the RSV *CP* gene by RT-PCR. (C) Time course of RSV symptom development in the WT and *NS3* OX or *mNS3* OX transgenic plants. Values represent the percentage of RSV-infected plants at various days post inoculation (dpi). Thirty plants were used for each treatment. **, *P* ≤ 0.01. Average (± SD) values from three biological replicates are shown.

Because NS3 induced miRNA accumulation along with a decreased antiviral defense response in rice, we speculated that NS3 may play a role in regulating viral pathogenicity. We used virus-free (mock) and viruliferous (RSV) planthoppers (*Laodelphax striatellus*) to inoculate the WT, and *NS3-* and *mNS3*-overexpression rice lines (*NS3* OX#1 and OX#7 and *mNS3* OX#1 and OX#4) and found that the *NS3* OX#1 and OX#7 lines, but not the *mNS3* OX#1 and OX#4 lines, were hypersensitive to RSV infection compared with WT plants, with most serious stunted and chlorisis phenotypes (Fig 6A). To examine whether the increased susceptibility of the *NS3* OX lines was due to the increased accumulation of RSV, we used RT-qPCR to measure the transcript levels of the RSV *CP* gene. We found that the expression of *CP* mRNA in the *NS3* OX#1 and #7 lines was much higher than that in the WT plants, with no obvious changes detected in the *mNS3* OX#1 and #4 lines when compared with the WT (Fig 6B). We also monitored differences in RSV infection rates among the WT and the *NS3* OX#1, *NS3* OX#7, *mNS3* OX#1, and *mNS3* OX#4 lines every 3 days until 21 dpi. These observations indicated that NS3 increased RSV pathogenicity in rice (Fig 6C and S3 Table). To test if NS3 OX plants are also more sensitive to infection with other viruses, we used *Rice ragged stunt virus* (RRSV), a member of the genus *Oryzavirus*, to infect the WT, and the *NS3* OX#1, *NS3* OX#7, *mNS3* OX#1, and *mNS3* OX#4 lines. The results showed that NS3 OX plant lines displayed a stunted phenotype (S4A Fig) and accumulated more RRSV *CP* genes (S4B Fig) compared with other rice plant lines.

## Discussion

**Fig 7:**
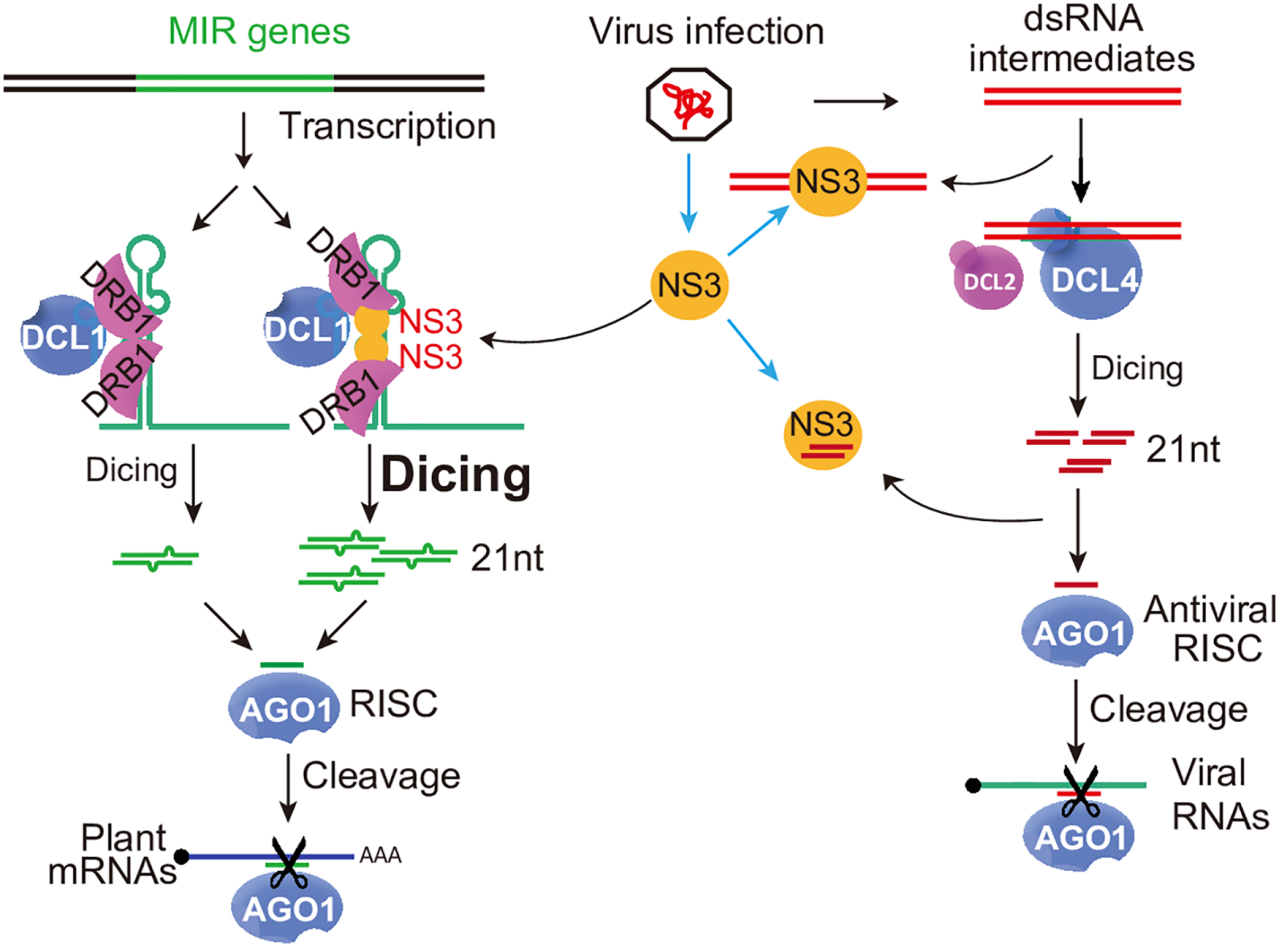
Proposed model. **NS3 represses the PTGS pathway.** Viral-derived long double-stranded RNAs (dsRNA) are processed into 21-nt siRNAs, the 21-nt siRNAs are loaded into AGO protein complex for cleavage of viral RNA genome RNAs. However, NS3 binds viral long dsRNA or siRNAs and inhibits siRNA production or the loading of siRNAs into AGO complex. Therefore, the PTGS pathway is blocked by NS3. **NS3 enhances the miRNA biogenesis pathway.** In mock-infected rice plants, a given pri-miRNA has a low probability of being recognized by dimeric DRB1 and then being processed into a mature miRNA by the processor complex. Consequently, the mature miRNA is insufficient to repress its target gene, which plays a crucial role in antiviral activity or development. Under RSV infection, pri-miRNA-bound NS3 interacts with DRB1 in sites required for DRB1 self-interaction and acts as a scaffold to regulate the association between DRB1 and the pri-miRNA. This increases the chance that this pri-miRNA will be processed into a mature miRNA. In addition, this compromises the expression of the target gene and enhances RSV pathogenicity.

RNA interference (RNAi) is a conserved and effective antiviral mechanism in plants and insects [44]. To counter the host’s antiviral defense mechanisms, viruses encode VSR (s) to interfere with the host’s RNAi. Most reported VSRs act to suppress the host’s RNA silencing system, such as by inhibiting viral RNA recognition, blocking dicing, suppressing assembly of the RNA-induced silencing complex (RISC), and preventing siRNA amplification [44–50]. RSV NS3, a reported RNA silencing suppressor, suppresses post-transcriptional gene silencing (PTGS) in *N*. *benthamiana* through its dsRNA binding ability [38]. Our previously obtained small RNA sequencing data of changes associated with RSV infection revealed that many miRNAs are induced by RSV infection [9,19,41]. Up to now, only a few studies have focused on how a virus hijacks the RNA silencing pathway to regulate miRNA accumulation and advance its own pathogenicity, with very limited reports focusing on how virus regulates miRNA processing. In the present study, we revealed that NS3 exploits OsDRB1, a key component of the D-body, to promote the processing of pri-miRNA along with the regulation of miRNA target gene expression (Fig 7). NS3 showed a weak interaction with OsDRB2 in the cytoplasm (S2G Fig). Since miRNA processing is occurred in the nucleus, we deduced that the association of NS3 and OsDRB2 would not affect NS3-mediated miRNA processing.

Previous studies showed that NS3 suppression of the PTGS pathway may depend on its siRNA and long dsRNA binding activity [37,38], which is different from the results of this study showing that NS3 enhanced the miRNA pathway through its function of bridging pri-miRNA and OsDRB1, a key player in miRNA processing complex. These two different functions may act in parallel. These results widen our knowledge of the molecular mechanisms underlying viral-host interactions.

Maturation of miRNAs is a complex process. miRNA-coding genes (*MIRs*) are transcribed by DNA-dependent RNA polymerase II (Pol II) and subjected to splicing and addition of a 5’ 7-methyguanosine cap and a 3’ polyadenylated tail [51]. Besides Pol II, many other Pol II-associated factors such as RNA-binding proteins PLEIOTROPIC REGULATORY LOCUS 1 and DAWDLE, CAP BINDING PROTEIN 20 and 80, SE, CAM33/XAP CIRCADIAN TIMEKEEPER and TOUGH have been reported to regulate miRNA transcription [52–59]. The structures of pri-miRNAs also affect pri-miRNA processing [60,61]. RSV infection results in the differential expression of pri-miRNAs in rice. For example, pri-miR168a and pri-miR399d were up-regulated, while other pri-miRNAs were down-regulated (Fig 2E). We revealed that NS3 mainly decreases the accumulation of pri-miRNAs, but the mechanism by which NS3 influences pri-miRNA expression levels remains unclear.

*De novo* smallRNA sequencing showed that NS3 and mNS3 have a strong influence on the accumulation of miRNAs, but not on other types of smallRNAs. The total number of miRNA reads in the *NS3* OX lines was higher than that in the WT, and the total number of miRNA reads in the *mNS3* OX lines was lower than that in the WT (S3B Fig), suggesting that NS3 may directly promote pri-miRNA recruitment by the processing complex. This hypothesis was supported by the finding that NS3 aids in *in vivo* pri-miRNA processing (Fig 3B). Further investigations showed that NS3 associated with OsDRB1a at sites required for OsDRB1a self-interaction (Fig 4F) and promoted the association of OsDRB1a with pri-miRNAs (Fig 5A). SmallRNA sequencing showed that the lengths and sequences of miRNAs were not changed by *NS3* or *mNS3* overexpression in rice (S1 Table). These results suggested that NS3 promotes recruitment of pri-miRNAs by the processor complex through an interaction with DRB1. The observation that NS3 overexpressing rice plants showed no notable developmental abnormities at vegetative stages under normal growth conditions may relate to the presence of target genes of miRNAs, regulated by NS3, in a complex genome, and a similar phenomenon was previously described [3,56,62]. A study in *Arabidopsis* showed that a set of miRNAs is regulated by DRB1 [63]. NS3 up-regulates several miRNAs in a DRB1 dependent manner (Fig 5D). These results may explain why NS3 could not enhance some miRNA which are DRB1-independent. But it’s still unknown how the preferential regulation of DRB1-dependent miRNA by NS3, we did not find similarities of sequences or secondary structure between precursors of DRB1-dependent miRNA.

Understanding the effects of NS3 of miRNA biogenesis will widen our understanding of the functions of virus-encoded proteins in regulating miRNA metabolism. Our findings of the association of NS3 with miRNA processing and accumulation provides insights into RSV pathogenicity and helps identify important targets for RSV infection, which can offer new strategies for the breeding or genetic engineering of RSV-resistant rice.

## Materials and methods

### Plant growth and virus inoculation

Rice (*Oryza sativa* spp. *japonica*) seedlings were grown in a greenhouse at 28–30°C and 60 ± 5% relative humidity under natural sunlight for 4 weeks. *Arabidopsis thaliana* plants were grown at 22°C under long-day conditions (16-h light/8-h dark). When the seedlings were about 10 days old, they were inoculated using viruliferous (RSV) or virus-free (mock) planthoppers (*L*. *striatellus*) at a ratio of four insects per plant for 72 h. After removal of the insects, the inoculated plants were returned to the greenhouse and monitored daily for the appearance of viral symptoms. The number of symptomatic rice plants of each line was recorded (Fig 6C).

### Vector construction and plant transformation

The Gateway system (Invitrogen) and the enzyme digestion connection method were used to make binary constructs. Binary gateway vectors pSAT4A-DEST-N(1–174)EYFP-N1, pSAT5A-DEST-C(175-END)EYFP-N1, pEarleyGate202, and pEarleyGate203 [64] were used for transient expression in *N*. *benthamiana* and stable transformation of *Arabidopsis*, while binary vectors pGEX-4T-1 and pCam2300:Actin1::OCS were used for *Escherichia coli* and stable rice transformations. Most cDNA and miRNA genes were cloned into pENTR/D and pEASY Blunt Zero vectors, and pENTR/D-mNS3 and mOsDRB1a clones were prepared using a Quik Change site-directed mutagenesis kit (Stratagene, La Jolla, CA, USA). After confirmation by sequencing, all clones were transferred to the appropriate destination vector by recombination using the Gateway LR Clonase II Enzyme mix (Invitrogen) or T4 DNA ligase (TransGen Biotech, Beijing, China). *Agrobacterium tumefaciens*-mediated rice transformation was carried out at Weiming Kaituo Co., Ltd. (Beijing, China). Transgenic *Arabidopsis* plants were selected by their resistance to Basta on soil.

### RNA extraction, quantitative RT-PCR, RT-PCR, and northern blot analysis

Total RNAs were extracted from plants using Trizol (Invitrogen). The extracted RNAs were treated with RNase-free DNase I (Promega, Madison, WI, USA) to remove DNA contamination and then reverse-transcribed with SuperScript III reverse transcriptase (Invitrogen) using oligo (dT) primers and a Mir-X miRNA first-strand synthesis kit (Clontech Laboratories). The resulting cDNAs were then used as templates for RT-qPCR and RT-PCR. RT-qPCR was performed using SYBR Green Real-time PCR Master Mix (Toyobo, Osaka, Japan). The rice *OsEF-1a* gene was detected in parallel and used as an internal control. Northern blot analyses were performed as previously described [65]. Briefly, RNA samples were separated by 15% denaturing gel electrophoresis and transferred onto Hybond-N+ membranes (Amersham, Fairfield, CT, USA). Membranes were UV cross-linked and hybridized to ^32^P end-labeled oligonucleotide probes. Sequences of primers used in RT–qPCR and northern blot assays are listed in S2 Table.

### Microscale thermophoresis assay

The Microscale Thermophoresis assays were performed as previously described [66,67]. GST-NS3, GST-OsDRB1a, and GST-mNS3 proteins were individually labeled with NHS red fluorescent dye according to the instructions of the RED-NHS Monolith NT Protein Labeling kit (NanoTemper Technologies GmbH, München, Germany). In protein and RNA interaction assays, the concentration of NHS-labeled protein was maintained at 100 nM, whereas RNA concentrations were gradient-diluted (20,000 nM, 10,000 nM, 5,000 nM, and then 2-fold dilutions until 10 nM). The RNA was denatured and annealed to form dsRNA, followed by addition of RNase inhibitor (1 U per group). After a short incubation, the samples were loaded into MST standard-treated glass capillaries. Measurements were performed at 25°C and LED and MST powers of 20% in buffer containing 20 mM Tris (pH 8.0) and 150 mM NaCl. The assays were repeated two times for each affinity measurement. Data analyses were performed using Nanotemper Analysis and OriginPro 8.0 software provided by the manufacturer.

### Electrophoretic mobility shift assays

The pGEX-4T-1-NS3, pGEX-4T-1-mNS3, and pGEX-4T-1-OsDRB1a constructs, as well as empty pGEX-4T-1 vectors, were individually transformed into *E*. *coli* Transetta (DE3) (Transgene, Beijing, China) with protein expression induced by IPTG. The soluble GST fusion proteins were extracted and immobilized onto glutathione sepharose beads (Amersham). RNAs were labeled with ^32^P–UTP using a T7 RNA Production system (Promega) and purified with a MEGAclear kit (Ambion, Waltham, MA, USA). Approximately 2,000 cpm of radioactive RNA was denatured and then annealed to form dsRNA. The dsRNA was incubated with individual GST fusion proteins in the presence of RNase inhibitor (1 U per group) for 30 min on ice. The mixtures were then run on an RNase-free native PAGE gel for 1 h at 4°C.

### Protein preparation and western blot analysis

The procedures used for protein extraction and western blotting have been described previously [5]. The following antibodies were purchased from commercial sources: anti-flag-peroxidase (Sigma, St. Louis, MO, USA), anti-Myc-peroxidase (Sigma), anti-Myc (Sigma), anti-FLAG (Sigma), anti-Actin (Easybio, Beijing, China), and anti-HYL1 (Agrisera AB Box 57, SE-911 21 Vännäs, Sweden).

### Small RNA sequencing

Small RNA cloning for Illumina sequencing was carried out at Bainuodacheng Co., Ltd (Beijing, China). Rice miRNA annotations were obtained from miRBase (http://microrna.sanger.ac.uk/sequences, Release 14). Statistical analysis of the small RNA data sets was performed using in-house Perl scripts [5].

### Bimolecular fluorescence complementation

BiFC assays were performed as previously described [22]. Briefly, we transformed the recombinant constructs into *A*. *tumefaciens* strain EHA105 and injected the *Agrobacterium* cultures into *N*.*benthamiana* leaves. After 3 days, we observed the tobacco epidermal cells with a confocal laser scanning microscope (LSM 710 NLO & DuoScan System, Zeiss).

### RNA immunoprecipitation

RNA immunoprecipitation was carried out essentially as previously described [68] using 1% formaldehyde-treated leaves, transiently expressed protein, and RNA for 72 h. RIP was carried out with the following modifications: nuclei were isolated, resuspended in high salt nuclear lysis buffer (20 mM Tris-HCl, pH 7.5, 500 mM NaCl, 4 mM MgCl_2_, 0.2% NP-40). The chromatin supernatant was diluted five times with dilution buffer (20 mM Tris-HCl, pH 7.5, 4 mM MgCl_2_, 0.2% NP-40) and immunoprecipitated using antibody. Pri-miRNAs were detected in the immunoprecipitates by RT-PCR using the artificial primers miR528-5p and -3p.

### Accession numbers

Sequence data generated in this study can be found in GenBank (https://www.ncbi.nlm.nih.gov/genbank/), the Rice Genome Database (http://rice.plantbiology.msu.edu/) and the *Arabidopsis* Information Resource (http://www.arabidopsis.org/) under the following accession numbers: *NS3* (AY284945.1), *AGO1a* (LOC_Os02g45070), *SULTR2;1* (LOC_Os03g09930), *CDS1* (LOC_Os07g46990), *LOC_Os08g45000*, *OsRFPH2-10* (LOC_Os06g06050), Os*DRB1a* (LOC_Os11g01869), Os*DRB1b* (LOC_Os12g01916), *OsDRB1c*(LOC_Os05g24160), *OsDRB2(*LOC_Os10g33970*)*, *OsDRB3 (*LOC_Os09g33460*)*, *OsDRB4 (*LOC_Os01g56520*)*, *AtHYL1* (At1g09700),*AtDCL1* (At1g01040), *AtSE* (At2g27100), and *AtCBP20* (At5g44200), *AtDRB2* (At2g28380), *AtDRB3* (At3g26932), *AtDRB4* (At3g62800), *AtDRB5* (At5g41070).

## Supporting information

S1 Fig Characterization of phenotypes and miRNA levels in RSV-infected and *NS3*-overexpressing *Arabidopsis*. (A) Detection of miR168 and miR395 in mock-infected and RSV-infected *Arabidopsis* plants by northern blotting. (B) Detection of miR168 and miR395 in *NS3* OX and *mNS3* OX transgenic *Arabidopsis* plants by northern blotting. (C) Phenotypes of RSV-infected *Arabidopsis* plants and detection of RSV genomic RNA1 and RNA3 accumulation by northern blotting. Scale bar = 1 cm. (D) Phenotypes of *NS3* OX and *mNS3* OX transgenic *Arabidopsis* plants. Scale bar = 1 cm. In (A) and (B), U6 served as a loading control, the expression levels in Col-0 mock-inoculated plants were set to a value of 1.0 and the expression in other plants are relative to this reference value.
(TIF)

S2 Fig Specific interaction of AtHYL1 and NS3 in the D-body and characterization of rice DRB1. (A) Results of a BiFC assay showing the specific interaction of NS3 and *Arabidopsis* HYL1 in the D-body. Scale bar = 0.1 μm. (B) Phylogenetic tree of all *Arabidopsis* and rice DRBs. (C) Detection of miRNA levels in an *OsDRB1*-knockdown line by small-RNA RT-qPCR. (D) Detection of OsDRB1 levels in the WT and an *OsDRB1*-knockdown line by western blotting. (E) BiFC assays test interaction of NS3 between OsDRB2, OsDRB3 or OsDRB4. Scale bar = 1 μm. (F) Amino acid alignment of OsDRB1a, AtHYL1 and dsRBD2 of AtHYL1. (G) BiFC assays of NS3-NS3, mNS3-mNS3, NS3-mNS3 and OsDRB1a-OsDRB1a interactions. Scale bar = 5 μm. The BiFC assay was conducted in *N*. *benthamiana* epidermal cell, mCherry is a nuclear marker fused with red florescence protein (RFP).
(TIF)

S3 Fig Characterization of the role of NS3 in miRNA accumulation and miRNA precursor binding. (A) Results of an EMSA showing that NS3 protein interacts with precursor miRNAs (pre-miR168a, pre-miR395d, pre-miR398a, pre-miR399d, and pre-miR528) whereas mNS3 does not. (B) Global effect of NS3 and mNS3 on small RNA accumulations.
(TIF)

S4 Fig Disease symptoms induced by RRSV infection in WT, *NS3* OX, and *mNS3* OX rice lines. (A) Images of whole plants showing stunted phenotypes of the wild-type, *NS3* OX#1, *NS3* OX#7, *mNS3* OX#1 and *mNS3* OX#4 rice plants. Scale bars = 15 cm. (B) Detection of the RRSV *CP* gene by RT-qPCR.
(TIF)

S1 Table Analysis of miRNA abundance in WT, *NS3* OX#1 and *mNS3* OX#1 by deep sequencing. (XLSX)

S2 Table Oligonucleotides used in this study. (XLSX)

S3 Table Statistics of rice plants exhibiting RSV disease symptom. (XLSX)
