# Supplementary figures and images for: Rice stripe virus NS3 protein regulates primary miRNA processing through association with the miRNA biogenesis factor OsDRB1 and facilitates virus infection in rice
Source: PLoS Pathog. 2017 Oct 4;13(10):e1006662. doi: 10.1371/journal.ppat.1006662 (PMC5658190; doi:10.1371/journal.ppat.1006662)

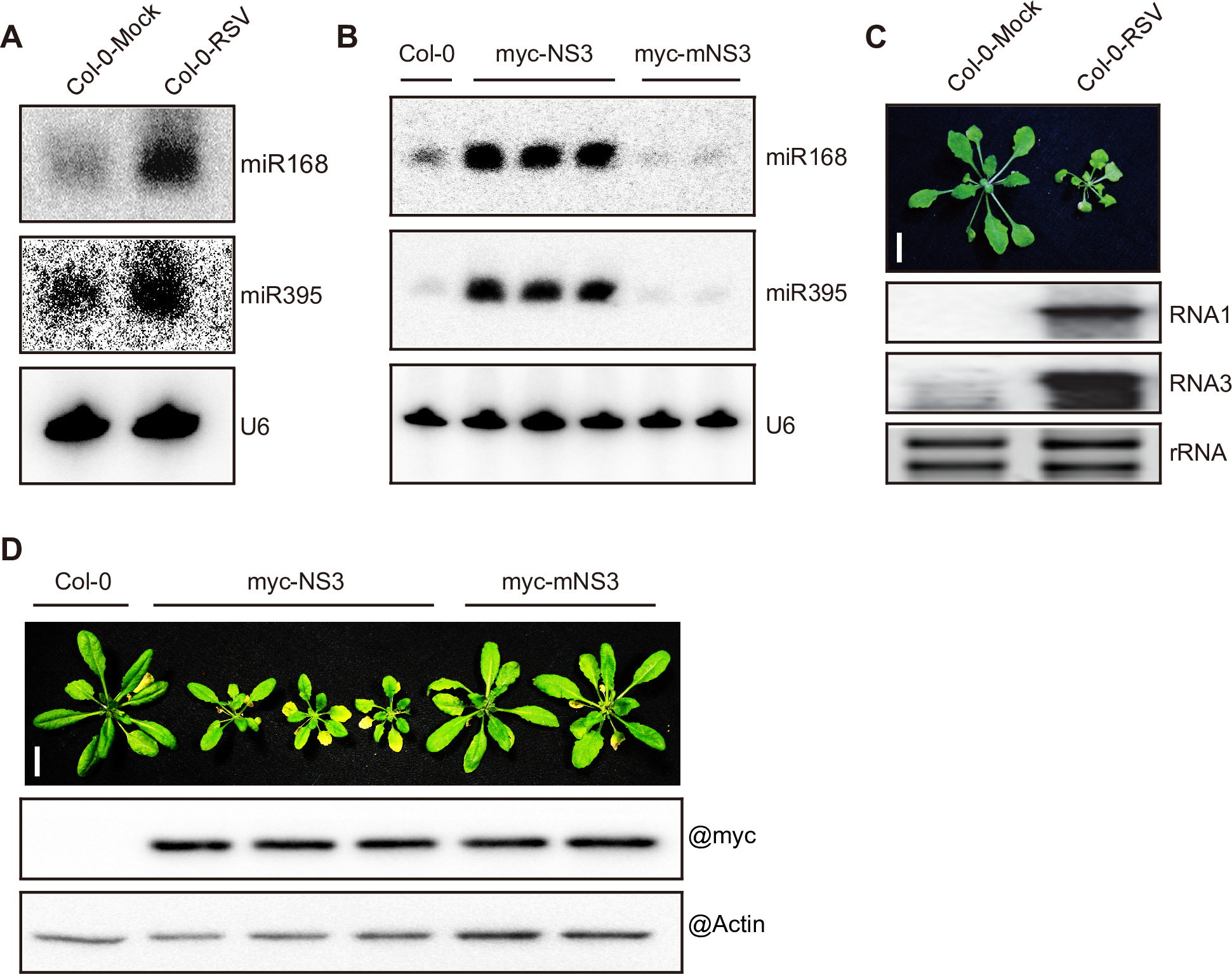

Supplement: S1 Fig — (A) Detection of miR168 and miR395 in mock-infected and RSV-infected Arabidopsis plants by northern blotting. (B) Detection of miR168 and miR395 in NS3 OX and mNS3 OX transgenic Arabidopsis plants by northern blotting. (C) Phenotypes of RSV-infected Arabidopsis plants and detection of RSV genomic RNA1 and RNA3 accumulation by northern blotting. Scale bar = 1 cm. (D) Phenotypes of NS3 OX and mNS3 OX transgenic Arabidopsis plants. Scale bar = 1 cm. In (A) and (B), U6 served as a loading control, the expression levels in Col-0 mock-inoculated plants were set to a value of 1.0 and the expression in other plants are relative to this reference value. (TIF) [file ppat.1006662.s001.tif]

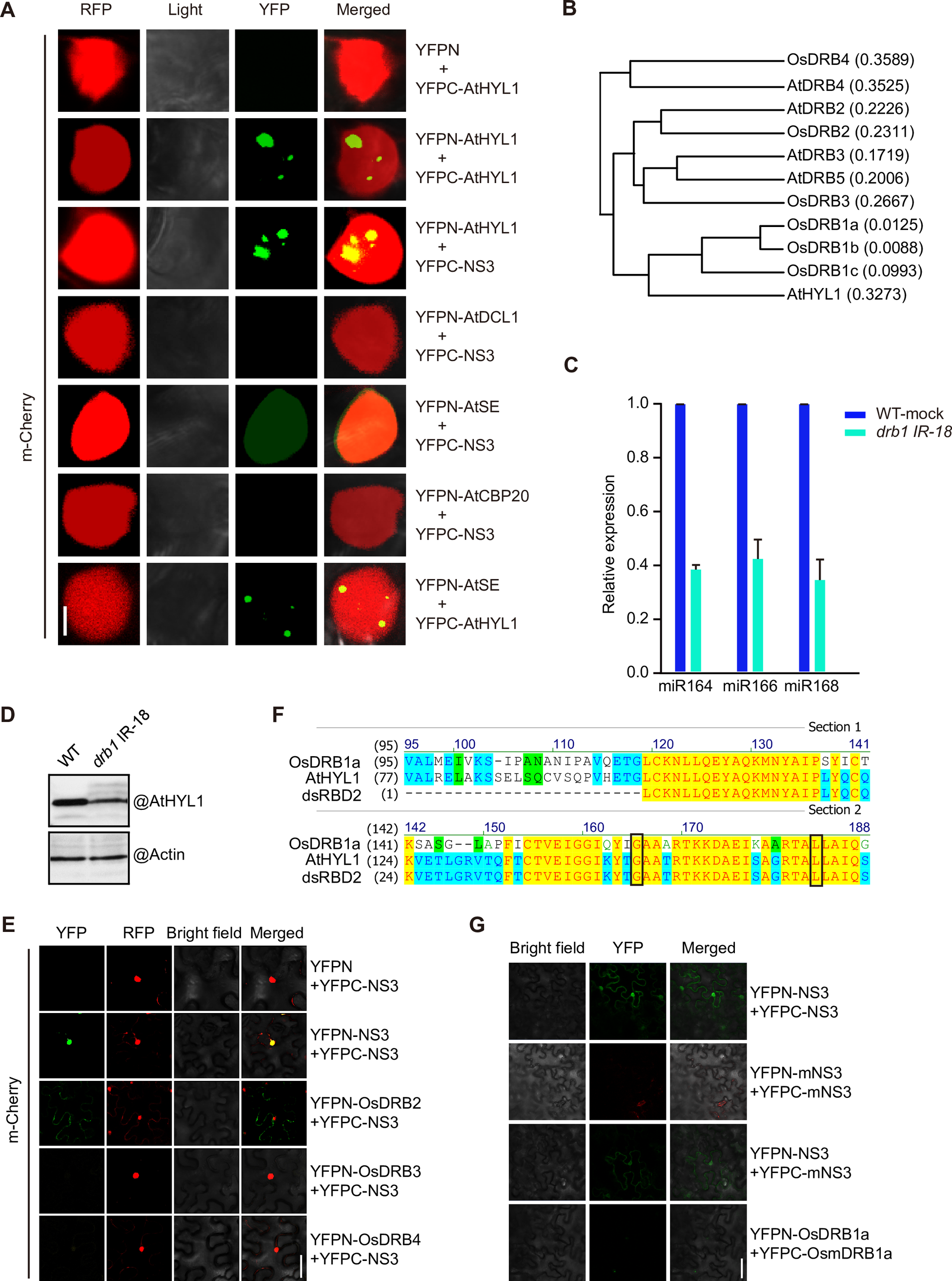

Supplement: S2 Fig — (A) Results of a BiFC assay showing the specific interaction of NS3 and Arabidopsis HYL1 in the D-body. Scale bar = 0.1 μm. (B) Phylogenetic tree of all Arabidopsis and rice DRBs. (C) Detection of miRNA levels in an OsDRB1-knockdown line by small-RNA RT-qPCR. (D) Detection of OsDRB1 levels in the WT and an OsDRB1-knockdown line by western blotting. (E) BiFC assays test interaction of NS3 between OsDRB2, OsDRB3 or OsDRB4. Scale bar = 1 μm. (F) Amino acid alignment of OsDRB1a, AtHYL1 and dsRBD2 of AtHYL1. (G) BiFC assays of NS3-NS3, mNS3-mNS3, NS3-mNS3 and OsDRB1a-OsDRB1a interactions. Scale bar = 5 μm. The BiFC assay was conducted in N. benthamiana epidermal cell, mCherry is a nuclear marker fused with red florescence protein (RFP). (TIF) [file ppat.1006662.s002.tif]

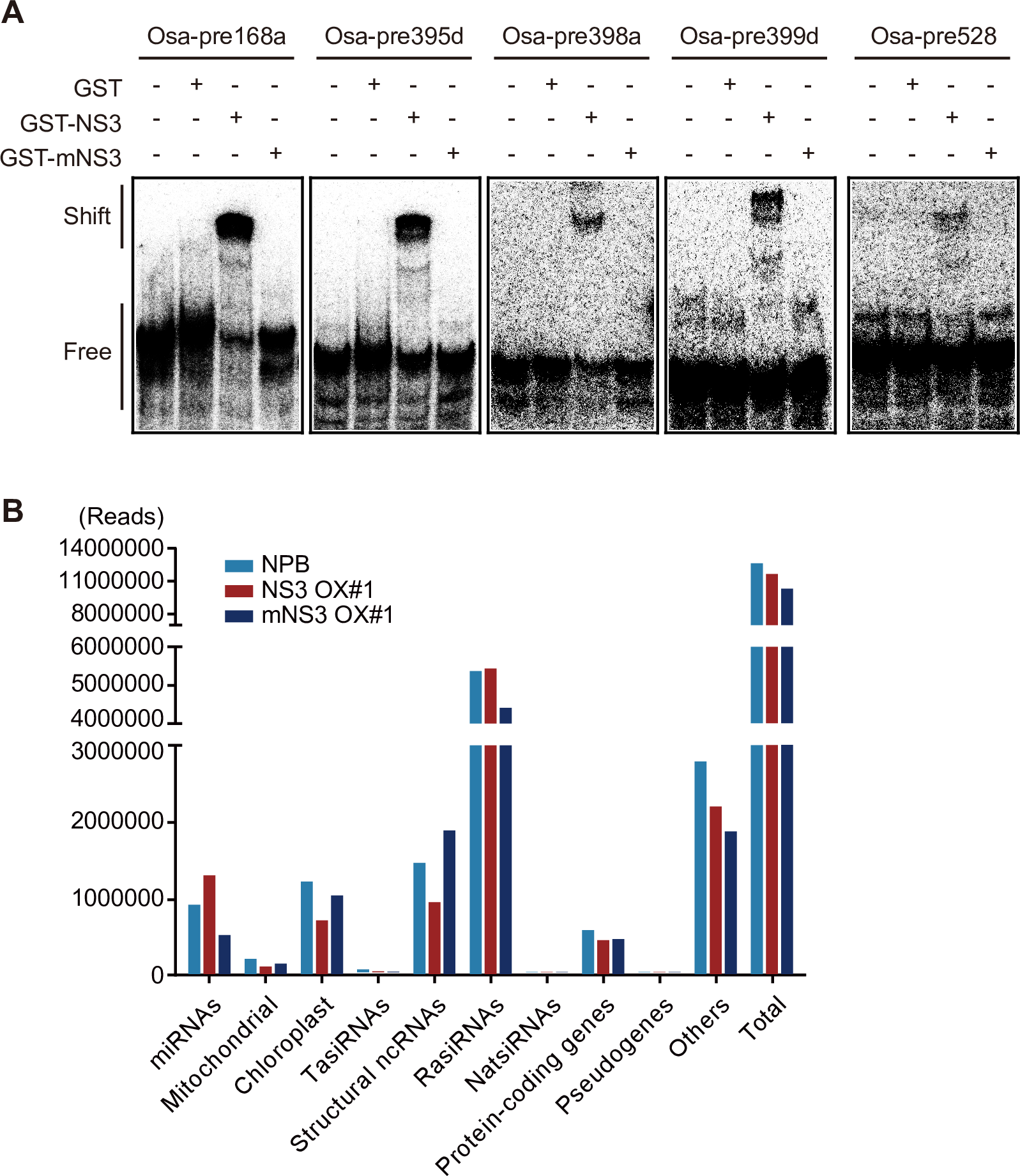

Supplement: S3 Fig — (A) Results of an EMSA showing that NS3 protein interacts with precursor miRNAs (pre-miR168a, pre-miR395d, pre-miR398a, pre-miR399d, and pre-miR528) whereas mNS3 does not. (B) Global effect of NS3 and mNS3 on small RNA accumulations. (TIF) [file ppat.1006662.s003.tif]

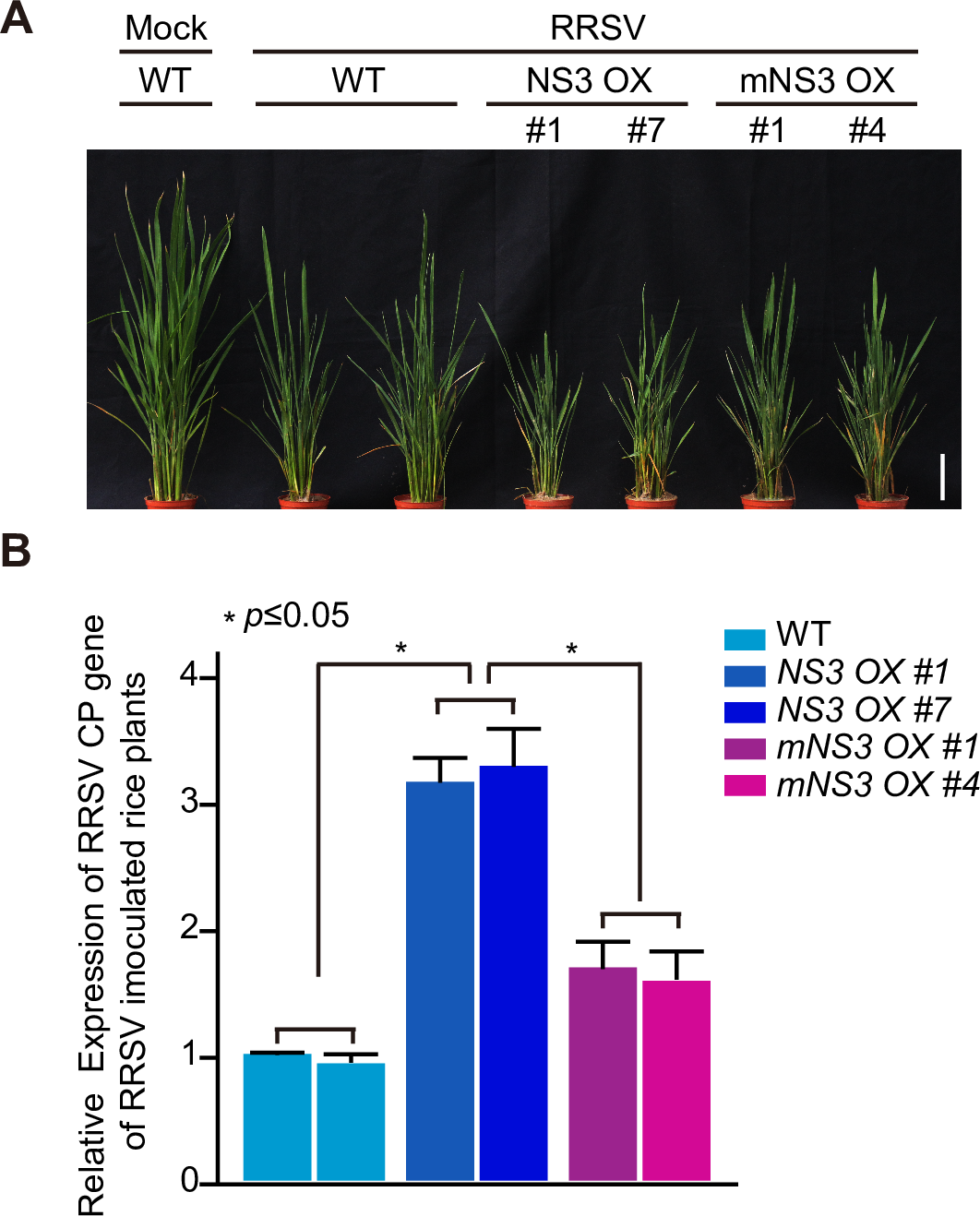

Supplement: S4 Fig — (A) Images of whole plants showing stunted phenotypes of the wild-type, NS3 OX#1, NS3 OX#7, mNS3 OX#1 and mNS3 OX#4 rice plants. Scale bars = 15 cm. (B) Detection of the RRSV CP gene by RT-qPCR. (TIF) [file ppat.1006662.s004.tif]
